# Supplementary material for: Trends in Women’s Empowerment and Their Association with Childhood Vaccination in Cambodia: Evidence from Demographic and Health Surveys (2010–2022)
Source: Vaccines (Basel). 2025 Dec 31;14(1):48. doi: 10.3390/vaccines14010048 (PMC12846650; doi:10.3390/vaccines14010048)
Supplement: Supplementary file 1 [file vaccines-14-00048-s001.zip › vaccines-4026887-supplementary.pdf]

Table S1. Schedule of childhood vaccines included in this analysis in the Cambodian National Immunization Programme (as of October 2024).

Vaccine (antigen)	Formulation in the national programme	Recommended ages (months)	Number of doses by 24 months
DTP	DTwP–Hib–HepB (pentavalent)	1.5, 2.5, 3.5 months	3
OPV	Oral poliovirus vaccine	1.5, 2.5, 3.5 months	3
13vPCV	13–valent pneumococcal conjugate	1.5, 2.5, 3.5 months	3
MR	Measles–rubella vaccine	9, 18 months	2

Note: Schedule adapted from the WHO regional analytical report [21].
